# Connexin and Pannexin hemichannels are regulated by redox potential

**DOI:** 10.3389/fphys.2014.00080

**Published:** 2014-02-25

**Authors:** Mauricio A. Retamal

**Affiliations:** Facultad de Medicina Clínica Alemana, Centro de Fisiología Celular e Integrativa, Universidad del DesarrolloSantiago, Chile

**Keywords:** carbon monoxide, nitric oxide, connexin, S-Nitrosylation, redox signaling

## Abstract

Connexins (Cxs) and Pannexins (Panxs) are two non-related protein families, having both the property to form hemichannels at the plasma membrane. There are 21 genes coding for different Cx based proteins and only 3 for Panx. Under physiological conditions, these hemichannels (Cxs and Panxs) present a low open probability, but when open, they allow the release of signaling molecules to the extracellular space. However, under pathological conditions, these hemichannels increase their open probability, inducing important lysis of metabolites, and ionic imbalance, which in turn induce the massive entry of Ca^+2^ to the cell. Actually, it is well recognized that Cxs and Panxs based channels play an important role in several diseases and -in many cases- this is associated with an aberrant hemichannel opening. Hemichannel opening and closing are controlled by a plethora of signaling including changes of the voltage plasma membrane, protein-protein interactions, and several posttranslational modifications, including protein cleavage, phosphorylation, glycosylation, hydroxylation and S-nitrosylation, among others. In particular, it has been recently shown that the cellular redox status modulates the opening/closing and permeability of at least Cx43, Cx46, and Panx1 hemichannels. Thus, for example, the gaseous transmitter nitric oxide (NO) can induce the S-nitrosylation of these proteins modulating in turn several of their properties. The reason is that the redox status of a cell is fundamental to set their response to the environment and also plays an important role in several pathologies. In this review, I will discuss how NO and other molecules associated with redox signaling modulate Cxs and Panx hemichannels properties.

## Connexin and pannexin hemichannels: general properties

Connexins (Cxs) are a family of proteins constituted by 21 members, while Pannexins (Panxs) have only 3 members. These two protein families are formed by fourth transmembrane domains, two extracellular loops, one intracellular loop and both the N and C-terminus located at the cytoplasm (Milks et al., 1988; Yeager and Gilula, 1992; Wang and Dahl, 2010). In spite of their topological similarities, Cxs and Panx share a very low homology in terms of their amino acidic sequence. However, in both cases the oligomerization of six subunits forms a channel frequently call “hemichannel.” But, it has been recently reported that Panx2 seems to form an octamer (Ambrosi et al., 2010), which would be the only exception to the hexameric hemichannels rule. Originally, the term “hemichannel” comes from the idea that the serial docking of two Cx-hemichannels form a gap junction channel (GJC). Therefore, a half GJC should be a hemi-channel. Even though the term hemichannel is widely used, some authors also used the word connexon to refer to these channels. As mentioned above, in the case of Panx, the term hemichannel is also used, but—as they seem not to form GJCs (Scemes et al., 2007) some authors have suggested to call them just channels (Sosinsky et al., 2011).

Cx- based GJC and hemichannels are formed by the same protein, but they have marked differences in terms of their cellular localization, opening and closing regulation and their roles in cellular processes. Thus, Cx-hemichannels are located at the plasma membrane zone that is not contacting with other cells, whereas GJC are located at the contacting zone. In the case of Panx- hemichannels, the history is more complex. There are a number of studies that support the idea that Panxs form only hemichannels *in vivo* (Scemes et al., 2007), probably because Panxs are glycoproteins and its posttranslational modification could interfere with the GJC formation (Penuela et al., 2007). However, Panx1 has been observed to form GJC in *Xenopus Laevis* oocytes heterologous expression system (Bruzzone et al., 2003), which indicates that at least Panx1, under certain conditions, can form intercellular channels. Due to their cellular localization, when hemichannels open the flow of molecules and ions between the intracellular compartment and the extracellular space is allowed. In particular, Cx- hemichannels have been associated with cell-cell autocrine/paracrine communication through ATP (Romanello and D'Andrea, 2001; Stout et al., 2002), glutamate (Ye et al., 2003), cyclic ADP-ribose [cADPR] (Bruzzone et al., 2001), cAMP (Valiunas, 2013) and PGE2 (release) and glucose uptake (Retamal et al., 2007a). Additionally, hemichannels are relevant players in calcium waves propagation (Cotrina et al., 1998; Stout et al., 2002), memory consolidation in the amygdala (Stehberg et al., 2012), cell proliferation (Song et al., 2010), cell migration (Cotrina et al., 2008), light processing by the retina (Kamermans et al., 2001; Vroman et al., 2013), among others. On the other hand, GJC allow the cells to share ions and metabolites directly (Sáez et al., 1989; Kam et al., 1998; Goldberg et al., 1999; Niessen et al., 2000). So far, Panx1 hemichannels have been shown to be permeable to ATP (Bao et al., 2004; Penuela et al., 2013) and, interestingly, it is probable to be the largest pore associated with the activation of the P2X7 receptor by extracellular ATP (Pelegrin and Surprenant, 2006; Iglesias et al., 2008). Thus, both Cx- and Panx- hemichannels are permeable to signaling molecules and, therefore, are associated with a great number of biological processes.

Taken together above evidence, it is now increasingly accepted that under physiological conditions Cxs- hemichannels can open, but with a low open probability (Contreras et al., 2003), which would be enough to participate in several cellular processes (Sáez et al., 2010; Rackauskas et al., 2010; Kar et al., 2012). However, under pathological conditions, Cx- hemichannels increase their overall activity most likely due to increasing the open probability and thus forming “leaky hemichannels” (Liang et al., 2005; Stong et al., 2006; Sánchez et al., 2010) and/or increasing their number at the plasma membrane (Retamal et al., 2006). This augmented hemichannel activity has been associated with an accelerated cell death in heterologous systems (Essenfelder et al., 2004; Gerido et al., 2007; Tong et al., 2011; Levit et al., 2012), supporting the idea that a low hemichannel activity can be related to several cell functions, but a high and/or uncontrolled hemichannel activity diminishes cell viability. Similarly, Panx- hemichannels also increase their activity under pathological conditions, thus Panx1 hemichannels increase their opening probability in cells metabolically inhibited (Domercq et al., 2010; Bargiotas et al., 2011), as well as under inflammatory conditions (Riteau et al., 2010; Orellana et al., 2011).

As presented before, maintaining a controlled opening/closing hemichannel is very important to preserve a normal cell function. Cx hemichannels are constantly under the control of several factors, including those acting intracellularly, as membrane potential (Ebihara, 2003; Bukauskas and Verselis, 2004; Kronengold et al., 2012), intramolecular interactions (Ponsaerts et al., 2010), pH (Peracchia, 2004) and posttranslational modifications, such as phosphorylation (Sáez et al., 1998; Lampe and Lau, 2000; Moreno, 2005), ubiquitination, SUMOylation, palmitoylation, caspasecleavage, S-Nitrosylation, hydroxylation and deamidation (reviewed by Johnstone et al., 2012; D'Hondt et al., 2013), as well as those acting extracellularly, such as Ca^2+^ and Mg^2+^ (Verselis and Srinivas, 2008; Bader et al., 2012). Similarly, Panx- hemichannels are also modulated by intracellular signaling molecules and posttranslational modifications, such as N-glycosylation in their extracellular loops [Panx1, Panx2, and Panx3, asparagine 254, 86, and 71, respectively] (Penuela et al., 2013). Notwithstanding, there is no confirmation yet that Panx are phosphoproteins; current evidence indicates that they can be so. Thus, a connection was observed between activation of kinases, such as Src (Weilinger et al., 2012) and Rho (Seminario-Vidal et al., 2009) and the Panx1 hemichannel opening. Finally, rises in the intracellular calcium concentration do increase the Panx1 hemichannel opening (Locovei et al., 2006). In conclusion, under physiological conditions, these molecular mechanisms act in combination to keep the hemichannels mostly closed. This mini review will focus on the effect of reactive oxygen species [ROS, i.e., nitric oxide (NO)] over the permeability and gating of Cxs and Panxs hemichannels.

## Connexin's hemichannels and their control by redox signaling molecules

Probably the first study suggesting that Cxs- hemichannels can be modulated by redox signaling molecules was the work performed by Contreras and his co-workers (2002). This work showed that rat's astrocytes in culture become permeable to fluorescent tracers Ethidium and Lucifer yellow, after being metabolically inhibited by 75 min with iodoacetic acid and antimycin A. This membrane permeabilization was blocked by Octanol, La^3+^ and 18α-glycyrrhetinic acid (AGA), three well known hemichannel blockers, and was not observed in astrocytes from mouse knockout for Cx43. These results indicate that metabolic inhibition induces the opening of Cx43 hemichannels. Additionally, it was observed that Trolox -a free radical scavenger- was very efficient in preventing the opening of hemichannels induced by metabolic inhibition (Contreras et al., 2002). This indicates that metabolic stress increases free radicals concentration by an unknown mechanism (at least in that date) inducing the Cx43 hemichannel opening. Interestingly, a progressive dephosphorylation of this protein was also observed in parallel with the metabolic inhibition progression. But even when Cx43 is dephosphorylated, which is a signaling to induce hemichannel opening (Bao et al., 2007), hemichannels were closed by Trolox indicating that the redox status of Cx43 is a control mechanism more important than dephosphorylation, at least in metabolic stress conditions. Then, Retamal et al. (2006) studied the molecular mechanism that control Cx43 hemichannel opening under metabolic inhibition. Here, it was observed that astrocytes under control conditions were permeabilized to Ethidium after being exposed to a nitric oxide (NO) donor (GSNO). This permeabilization was reverted by DTT, indicating that NO induces the opening of Cx43 hemichannels by a mechanism dependent of S-nitrosylation (Retamal et al., 2006). Because of the above, the effect of DTT was tested in astrocytes under metabolic inhibition, and it was observed that DTT was able to block the entry of Ethidium into the cells. A similar result was observed when a reduced gluthation that is able to cross the plasma membrane (GSH-EE) was added to the bath solution, but this reduction was not observed when GSH (which does not cross the membrane) was added (Retamal et al., 2006). This indicates that the hemichannel blocking induced by reducing agents is due to reduction of the oxidized intracellular cysteines (Cys). At this point, it was not yet determined which Cx43 Cys were modified by NO, but it was demonstrated that Cx43 is S-nitrosylated by GSNO and also by metabolic inhibition, thus changing some properties of these channels.

Then, we studied the effect of redox molecules in Cx43 expressed in HeLa cells (Retamal et al., 2007b). First, we studied the effect of GSNO on these hemichannels, and unexpectedly no changes were observed in the activity of Cx43 hemichannels (data not published). However, it was evident that a reducing agent such as DTT presents a robust activation of these hemichannels, which was observed as an increase of dye uptake and by electrophysiology experiments. This indicates that HeLa cells somehow keep Cx43 hemichannel's Cys groups oxidized and, therefore, susceptible to be reduced by reducing agents such as dithiothreitol (DTT). Now, in astrocytes, oxidative stress open hemichannels and in HeLa cells reducing agents induce the opening of hemichannels. How can this be possible? To answer this question, Retamal et al. (2007b) added DTT to cells under metabolic inhibition at different times and it was observed that, when DTT was added before 20 min of metabolic inhibition, it induced an increase in Ethidium uptake. Moreover, when DTT was added around 30 min of metabolic inhibition no clear effect was observed, but when added after 40 min, it induced closure of Cx43 hemichannels. These data suggest that even when Cx43 hemichannels are affected by the cellular redox potential, the net effect is probably going to depend upon some other factors, such as the phosphorylation/dephosphorylation balance. Thus, it is necessary to elucidate the possible cross-talk between phosphorylation, pH, and membrane potential within the final effect induced by oxidation. Experiments based on this line of thought have been published (De Vuyst et al., 2009), but more research is still needed.

As mentioned before, Cx43 hemichannels are sensitive to redox potential, but until now there is no evidence showing which is or are the Cys groups that can be modulated by NO or other free radicals. It has been suggested that Cys271 could be a good candidate, because this Cys has been shown to be S-nitrosylated in GJC formed by Cx43 in endothelial cells (Straub et al., 2011). Interestingly, this posttranslational modification seems to decrease the permeability to IP_3_ through Cx43 GJC, and it is constantly modulated by the S-nitrosogluthathion reductase (Straub et al., 2011).

In another experiment performed in astrocytes in culture, the effect of proinflammatory cytokines on GJC and hemichannels were analyzed. Under these conditions, GJC between astrocytes were found to be closed, measured as a decrease in the intercellular transference of Lucifer yellow, but in parallel it was observed an increase in Ethidium uptake, which would suggest the opening of Cx43 hemichannels (Retamal et al., 2007a). The increase of hemichannel activity was prevented by L-name (an inhibitor of the nitric oxide syntase) and also by DTT. But, the decrease of cell coupling was affected neither by DTT nor by L-name (Retamal et al., 2007a). Above findings suggest that Cx43 hemichannels are more sensitive to changes in the redox potential than GJC and/or the modifications in different Cys groups show differences in terms of sensitivity to reducing agents. Similar results have been observed in ryanodine receptors, where different Cys groups are differentially oxidized (i.e., by *S*-nitrosylation, *S*-glutathionylation, and disulfide oxidation) and induced different modification to channel properties (Aracena-Parks et al., 2006). On the other hand, the increase of Cx43 hemichannels activity in astrocytes induced by NO can lead to a massive neuronal death due to considerable efflux of glutamate from astrocytes (Froger et al., 2010) and the inhibition of Cx43 hemichannels by synthetic cannabinoids was neuroprotective (Froger et al., 2010). Therefore, more studies are necessary to find out the effect of other oxidizing agents that affect Cys groups, such as oxidized glutathione, which will help to understand the role of redox potential as controller of hemichannels in pathological and also physiological conditions.

It has been recently reported that Cx46 is also modulated by the NO donor GSNO (Retamal et al., 2009). In this work, GSNO was shown to induce changes in Cx46 hemichannel opening and closing kinetics, current—voltage relationship and also induces the appearance of a current relaxation at voltages over +40 mV. All of them are reverted by DTT, suggesting that GSNO can induce the oxidation of Cx46 in Cys groups. These modifications were not observed in a Cx46 without intracellular Cys (Cx46C3A), thus suggesting that some of the two intracellular Cys are S-nitrosylated. Additionally, it was observed that GSNO induces a slight (but statistically significant) decrease in the hemichannel permeability to large molecules (i.e., Lucifer yellow) (Retamal et al., 2009). To date, it has not been possible to elucidate which is/are the Cys involved in this phenomenon. However, these data seem to be relevant to understand the role of free radicals as initiators and/or enhancers of lens opacity (disease commonly known as cataracts) (Berthoud and Beyer, 2009; Retamal et al., 2011).

A recent study has shown that Cx32, Cx37, and Cx40 are also affected by NO (Figueroa et al., 2013). In this work, it was determined that NO induced the opening of Cx37 and Cx40, whereas it induced the closure of Cx32. In all cases (including Cx43), hemichannels were permeable to NO (Figueroa et al., 2013). This work suggests that hemichannels formed by Cx32, 37, 40, and 43 could be good pathways for the diffusion of NO between endothelial cells and smooth muscle cells.

**Figure 1:**
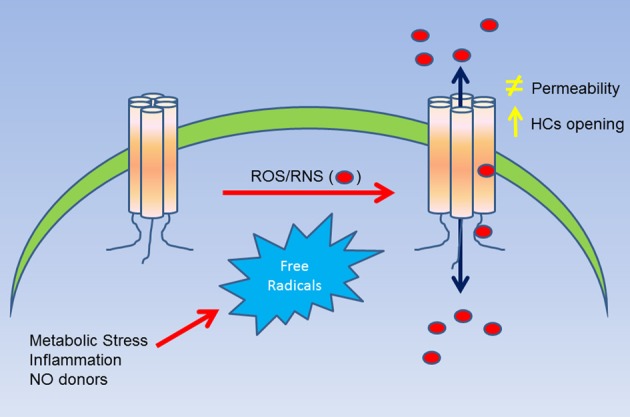
**Summary of the effect of ROS/RNS over Cxs hemichannels.** A cellular stress (i.e., metabolic inhibition) induces an increase of ROS/RNS production, which in turn, can affect directly Cx hemichannels (as observed in Cys271 of Cx43). This molecular modification can induce and increase in the open probability of hemichannels and/or changes in their permeability to large molecules. Additionally, the posttranslational modification induced by ROS/RNS can also lead to an increase of the permeability of nitro oxide and possibly of other free radicals as well.

Finally, hemichannel opening has been observed in other models of oxidative stress, such as cadmium-induced oxidative stress (Fang et al., 2011) and smoking-induced cell injury (Ramachandran et al., 2007). In the work of ramachandran, they observed that CSE (cigarette smoke extract) and h_2*o*2_ were able to cross the plasma membrane through the open hemichannels. This is consistent with the work of Figueroa et al. (2013), suggesting that hemichannels are not only affected by free radicals but also are permeable to them (Figure 1). Interestingly, in 2012 a mutant of Cx31 (Cx31R42P) was reported to produce hemichannels with a gain in activity, which induced cell death (Chi et al., 2012). This cell death was prevented by hemichannel blockers, increasing the extracellular concentration of Ca^2+^, and by the ROS scavenger butylated hydroxyanisole (BHA) (Chi et al., 2012). In this work, authors suggested that this mutation somehow would induce an increase in the ROS production by the cells. Moreover, as Cx31 has several cys groups in their c-terminus, it is possible that these ROS are oxidizing Cx31 hemichannels, which in turn would induce the hemichannel opening, similarly to previous observation made in Cx43 hemichannels expressing cells (Contreras et al., 2002; Retamal et al., 2006).

To date, no direct evidence is available to support the possibility that gsgg, h_2*o*2_, carbon monoxide (co), hydrogen sulphide (h_2*s*_) or any other oxidant molecules may affect the properties of cx hemichannels. Therefore, it is absolutely essential to investigate the effect of these molecules in hemichannels formed by cxs *in vitro* and *in vivo* in order to better understand the role of ROS in hemichannel function.

## Pannexin's hemichannels and their control by redox signaling molecules

Panx1 is expressed in several types of cells (Bruzzone et al., 2003). As observed for Cx43 hemichanels in astrocytes, neurons under ischemic-like conditions open their Panx1 hemichannels (Thompson et al., 2006), phenomenon that was inhibited by DTT and L-NAME, and suggesting that NO is involved in neuronal Panx1 hemichannel opening (Zhang et al., 2008). In the same line of evidence, Panx1 hemichannels are closed by reducing agents (Bunse et al., 2009). Accordingly, mutation of Cys 40 and 346 prevent the GSNO induced S-nitrosylation of this protein and also prevent Panx1 hemichannels opening (Lohman et al., 2012). The substitution of Cys40 or Cys346 by Serine induced the appearance of hemichannels constitutively open (Bunse et al., 2009, 2010). In contrast, mutation of Zebra fish Cys282 to tryptophan (C282W) reduced the hemichannel activity (Prochnow et al., 2009). Above results suggest that these Cys groups are probably the redox potential sensor in this protein, and they can vary depending on the species. Interestingly, it has been recently proposed that Panx1 hemichannels can help NO to cross the plasma membrane (Campanucci et al., 2012), phenomenon recently shown for Cx- hemichannels (Figueroa et al., 2013). Taking into account all the evidence, it is possible to suggest that under physiological conditions, intracellular reduced glutathione (GSH) keeps Panx1 hemichannel closed, but when the free radical concentration increases, due to (for example) metabolic stress, Panx1 hemichannels become open. In summary, Panx1 is affected by ROS, but the exact molecular mechanism is not completely understood yet. At present, there are no data showing the effects of ROS upon the activity/properties of hemichannels formed by Panx2 and Panx3.

## Redox signaling mediates hemichannels function in pathological conditions

The role of Cxs and Panx hemichannels on pathological conditions has been extensively reviewed (Bennett et al., 2012; Orellana et al., 2013). Therefore, this section will be focused on the role of redox signaling as intermediary of cellular response mediated by Cxs and Panx hemichannels in unhealthy cells. It is well accepted that under pathological conditions there is an increase in ROS/RNS production, which can affect different proteins through posttranslational modifications (Kolluru et al., 2013; Nakamura et al., 2013), thus, affecting diverse cellular functions (Gaston et al., 2003). In this context, an enhanced production of for example NO will lead to an increased hemichannel opening, which in turn will have several consequences in the cellular function as discussed before. Now, it is important to mention that the net effect of NO on hemichannels is going to depend on the level of NO produced. Thus, moderated levels of NO will induce a moderate hemichannel opening, which will have a profound effect in the autocrine/paracrine communication. This is because it is known that Cx43 hemichannel opening allows the release of signaling molecules to the extracellular space such as ATP (Stout et al., 2002), which is a well-recognized molecule involved in inflammatory processes (Eltzschig et al., 2012), mainly through P2X7 activation (Arulkumaran et al., 2011). Thus, hemichannel opening induced by NO could be a key point in several inflammatory processes. On the other hand, a large NO production may induce a massive hemichannel opening that is a signal that enhances and/or accelerates cell death (Retamal et al., 2006; Sáez et al., 2010). Therefore, depending on the NO production, the hemichannel activity may lead to a wide range of responses, from tissue inflammation to massive cell death. Table 1 summarizes the effect of ROS/RNS over Cx and Panx hemichannels.

**Table 1:** **List of all Cxs and Panx hemichannels that are known to be affected by changes of cellular redox potential**.

**Connexin/Pannexin**	**Molecule/Condition**	**Experimental model**	**Amino acid modified**	**Effect**	**Ref**
Cx32	NO donor	Fleta		Decrease hemichannel opening	Figueroa et al., 2013
Cx37	NO donor	Hela		Increase hemichannel opening	Figueroa et al., 2013
Cx40	NO donor	Hela		Increase hemichannel opening	Figueroa et al., 2013
Cx43	Metabolic Inhibition!	Astrocytes		Increase hemichannel opening	Contreras et al., 2002
	NO donor				
	NO donor	Astrocytes		Increase hemichannel opening	Retamal et al., 2006
	DTT	HeLa		Increase hemichannel opening	Retamal et al., 2007b
	Inflammation like condition	Astrocytes		Increase hemichannel opening	Retamal et al., 2007b
	NO donor	Endothelial Cells	C271-S-nitrosylation	Decrease IP3 permeability of GJCs	Straub et al., 2011
	smoking-induced cell Injury	N2A		Increase hemichannel opening	Ramachandran et al., 2007
	Cadmium-induced oxidative str.e.	Fibroblast		Increase hemichannel opening	Fang et al., 2011
Cx46	NO donor	Xenopus Oocyte		Modify VoItaje sensitivity and opening-closing kinetics	Retamal et al., 2009
Panxl	Ischemic-like conditions	Neurons		Increase hemichannel opening	Thompson et al., 2006
	Ischemic-like conditions	Neurons		Increase hemichannel opening	Zhang et al., 2008
	TCEP	N2A		Decrease hemichannel opening	Bunse et al., 2009
	NO donor	HEK293T	Cys 40 and 346—S-nuosyla:lon	Decrease hemichannel opening	Lohman et al., 2012

This table specifies the model in which the experiments were performed, the observed effects on hemichannel activity and which Cys group was modified.

**Figure 2:**
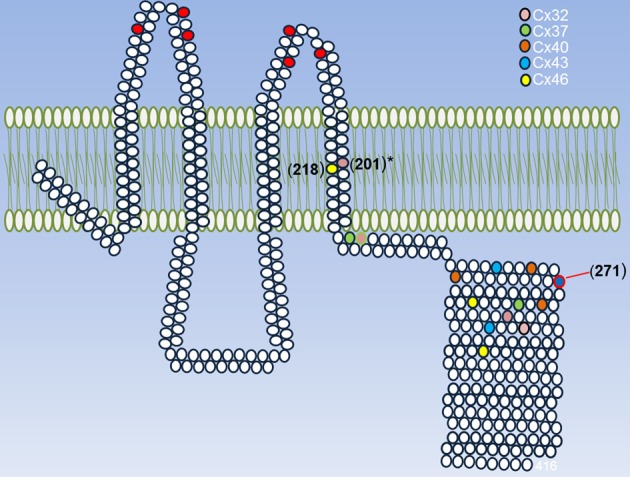
**Summary of Cys that can be modified by ROS/RNS.** A representative Cx is shown and its conserved extracellular Cys is highlighted (red circles) and each cysteine present in Cxs 32, 37, 40, 43, and 46 are shown in different colors. These Cxs were chosen because they are sensitive to redox potential. The exact position for each Cys was taken according to their last aa in the TM4. The asterisk in Cys201 of Cx32 indicates that their mutations induce the appearance of a disease and the Cys 271 of Cx43 is the only one that has proved to be S-nitrosylated.

Another important issue about redox control of hemichannel activity in physiological and pathological conditions is that at least Cxs that seem to be sensitive to NO. As connexins, ryanodine receptors show several Cys groups and have been shown that they can be differentially modify by NO (S-nitrosylation) or GSSG (S-gluthationylation) (Aracena-Parks et al., 2006). These differences in Cys modifications depend on their microenvironment in which a given Cys is located (Lam et al., 2010). Similarly, it can be postulated that different Cys in different regions on a given Cxs or Panx, could be affected by NO or GSSG in a different way and that may induce different changes in the hemichannels properties. Therefore, it is very important to study the effect of other oxidant molecules, such as H_2_S, CO, GSSG on hemichannel activity and test which are the Cys that are modified by these molecules. Also, it would be interesting to study if these modifications are present in proteins expressed in cells under pathological conditions. Following this line of evidence, our research group found that in rat's lenses that present cataract, Cx46 is S-nitrosylated, which suggests that at least this Cxs is modified by NO in pathological conditions, hence validating the data obtained *in vitro*. In Figure 2, it is observed all Cys groups present in Cxs sensitive to redox potential that are located in the TM4 and mostly in a region between 20 and 70 aa far from the beginning of the C-terminus. Although there are no conserved Cys, it is possible to postulate a zone sensitive to ROS/RNS. Actually, a Cys271 of Cx43 (which is already S-nitrosylated) is found in this putative zone. Cys in TM4 could be important redox sensors because rCx46 truncated C-terminus (Cx46ΔCT, which lacks Cys group in their C-terminus) and hCx46WT (which has only a Cys in TM4) are sensitive to nitric oxide (personal observation). Cys in a similar position—Cys201 (Morlé et al., 2000) and their homologous in Cx26—Cys202 (Sillén et al., 1998) -when mutated- induce the appearance of X-linked Charcot-Marie-Tooth disease and deafness, respectively. It is important to emphasize that there are Cxs with differences between their expression of Cys in their C-terminus (Table 2, Cx40 and Cx46), but there are others Cxs presenting important homologies (Table 2, Cx32 and Cx37) (Table 2).

**Table 2:**
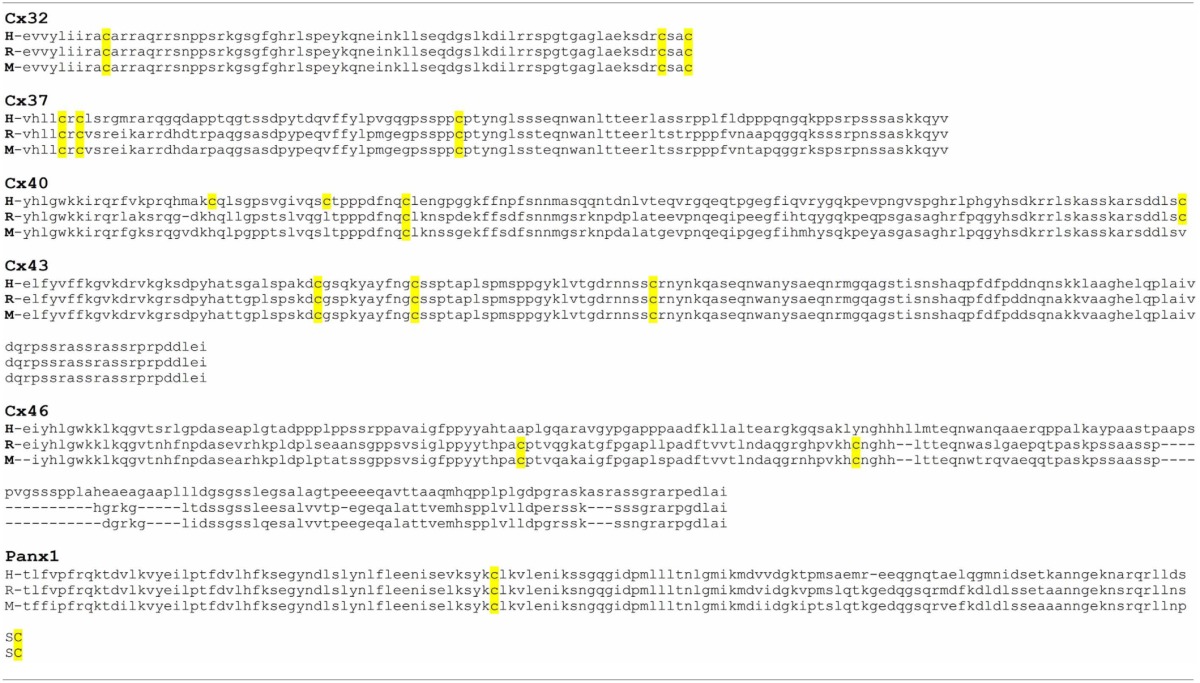
**Alignment of C-terminus of human, rat and mouse Cxs and Panx modified by nitric oxide**.

Cys in each C-terminus protein is shown in yellow. Note that all Cys are conserved in Cx32 in spite of large differences in Cx46. Human Cx46 has no Cys in C-terminus, but a Cys is present in position 218, in transmembrane 4. It has been reported that Cys located in the membrane can be S-nitrosylated (Leclerc et al., 2006). Therefore, the possibility that hCx46 can be redox sensitive cannot be ruled out.

NO is a highly reactive gas that participates in several physiological and pathological processes (Wang et al., 2010; Anand and Stamler, 2012; Nakamura et al., 2013). Recently, this gaseous transmitter has been shown to be permeable through hemichannels formed by Cxs (Figueroa et al., 2013). Considering that Cxs and Panx hemichannels are modulated by NO (Retamal et al., 2006, 2009; Lohman et al., 2012), it is possible to suggest that under pathological conditions hemichannels are playing an amplifier role of signaling pathways activated by NO, and –additionally- allowing NO to diffuse easily and widely in a tissue. On the other hand, it is known that there is a good correlation between intracellular Ca^2+^ levels and NO production. Recently, it has been demonstrated that Cx26 and Cx43 hemichannels are permeable to this cationic divalent ion (Sánchez et al., 2010; Schalper et al., 2010; Fiori et al., 2012). Thus, under pathological conditions where hemichannels increase their open probability, hemichannels can induce intracellular Ca^2+^increase, which in turn can activate intracellular pathways, such as caspases (Ishiura, 1981) and NO production (Schmidt et al., 1992). In this context, it has been recently shown that hemichannels participate in the NO production in injured endothelial cells of aorta (Berra-Romani et al., 2013). Therefore, several cellular responses induced by redox molecules (oxidant or reducing ones), such as; increased intercellular communication mediated by ATP and activation of Ca^2+^ dependent intracellular signaling pathways, can be mediated, at least in part, by inducing hemichannel opening. Finally, since the intracellular Ca^2+^ concentration is a good indicator of cellular health (Rasmussen et al., 1990), hemichannel opening under pathological conditions can set not only the cell response to an injury, but also determine the final destination of a cell.

## Conclusions and perspectives

For many years, much research has been performed to elucidate the molecular mechanism involved in the control of opening and closing of Cx and Panx- hemichannels, while the exact molecular mechanism has not yet been resolved. The most studied are probably: changes of membrane potential, phosphorylation, pH and extracellular divalent cations. Recently, another control mechanism has been proposed and it seems to affect several different types of Cxs and- at least- Panx1 hemichannel function. This new control mechanism is linked to changes of redox potential. Presently, the effect of NO upon Cx- and Panx hemichannels has been the most studied. However, there are other important free radicals that act in both pathological and physiological conditions. I would like to point out that carbon monoxide (CO) and hydrogen sulphide (H_2_S) are emerging as important gaseous transmitters with relevant roles in cell physiology (Wilkinson and Kemp, 2011; Ju et al., 2013). Thus, future lines of research will be probably focused on: (1) the effect of CO and H_2_S upon Cxs and Panx- hemichannels and GJCs; (2) Which is or are—at molecular level- the redox sensor(s) in different Cxs and Panxs, (3) Which are (if any) the hypothetical interactions between the redox sensor and the well-recognized slow and fast gating of Cxs hemichannels, and (4) which are the modifications on the structure of the Cxs in terms of folding, protein-protein bindings and intramolecular interaction.

### Conflict of interest statement

The author declares that the research was conducted in the absence of any commercial or financial relationships that could be construed as a potential conflict of interest.

## Abbreviations

CO: Carbon Monoxide
NO: Nitric oxide
Cx: Connexin
GJC: Gap junction channel
Panx: Pannexin
Cys: Cysteine.
